# Functional Connectivity Methods and Their Applications in fMRI Data

**DOI:** 10.3390/e24030390

**Published:** 2022-03-11

**Authors:** Yasaman Shahhosseini, Michelle F. Miranda

**Affiliations:** Department of Mathematics and Statistics, University of Victoria, Victoria, BC V8W 2Y2, Canada; yshahhosseini@uvic.ca

**Keywords:** fMRI, functional connectivity, brain network, Human Connectome Project, statistics

## Abstract

The availability of powerful non-invasive neuroimaging techniques has given rise to various studies that aim to map the human brain. These studies focus on not only finding brain activation signatures but also on understanding the overall organization of functional communication in the brain network. Based on the principle that distinct brain regions are functionally connected and continuously share information with each other, various approaches to finding these functional networks have been proposed in the literature. In this paper, we present an overview of the most common methods to estimate and characterize functional connectivity in fMRI data. We illustrate these methodologies with resting-state functional MRI data from the Human Connectome Project, providing details of their implementation and insights on the interpretations of the results. We aim to guide researchers that are new to the field of neuroimaging by providing the necessary tools to estimate and characterize brain circuitry.

## 1. Introduction

Functional magnetic resonance imaging (fMRI) techniques have emerged as a powerful tool for the characterization of human brain connectivity and its relationship to health, behavior, and lifestyle [1]. The fMRI measurements comprise of an indirect and non-invasive measurement of brain activity based on the blood oxygen level dependent (BOLD) contrast [2]. Compared to alternative brain imaging modalities such as positron emission tomography (PET) and eletroencephalography (EEG), fMRIs are non-invasive and have a high spatial resolution, which makes them a popular choice in large brain imaging studies. An example of such studies is the Human Connectome Project that aims at understanding the underlying function of the brain by describing the patterns of connectivity in the healthy adult human brain [3].

There are mainly two goals in such studies: first, to identify location signatures in the brain that respond to external stimuli, and second, to identify brain space–time association patterns that emerge when the brain is either at rest or performing a task. These association patterns are measures of co-activation in functionally connected time series of anatomically different brain regions, known as functional connectivity [4,5]. There is evidence that individual differences in these connectivity patterns are responsible for important differences in cognitive and behavioral functions. Therefore, understanding these patterns can play an important role in predicting the early onset of neurodegenerative diseases and in monitoring disease care and treatment [6,7].

Functional MRI data is often high-dimensional and consists of images of 3D brain volumes collected over a period of time. In a typical study, the number of voxels $N_{v}$ is in the hundred of thousands, and the number of time points *T* is in the hundreds. Therefore, estimating the $N_{v}\times N_{v}$ correlation matrix of voxelwise spatial connectivities is challenging and requires a few strategies and assumptions. A simple technique is to first pre-specify regions called *seeds* and then compute the cross-correlation of seeds and the functional time series of every other voxel in the brain. This *seed-based approach* became popular due to its straightforward calculation and interpretation. Seeds can vary in size and be as small as a single voxel. If the seed is a region, it is customary to average the time courses of the region and use that as the reference time course to be correlated with all the other voxels. In order to improve scalability, it is also common to first parcellate the brain into small regions and use the average time series of these regions to estimate the networks. The seed-based method can be a helpful resource when comparing patterns of neuropathologies and the normal brain. For example, ref. [8] uses this method to show that connectivity between the hippocampus and other brain regions change with the early signs of Alzheimer’s disease when compared to control subjects. Despite the popularity of these approaches, there are various criticisms to the method. First, by focusing on pre-determined seeds, potential patterns that emerge in different brain regions are ignored [9]. Second, the method neglects the variability across voxels by averaging the time series in the ROIs. Third, the approach computes correlations between pairs of nodes and ignores the potential influence of other nodes in the entire network.

In contrast to region pre-specification, dimension reduction approaches characterize the spatial and/or temporal connectivity patterns by representing the data through a small number of latent components [10]. Principal component analysis (PCA) and independent component analysis (ICA) are the two most common of these methods. Both methods project the high-dimensional imaging data into a low-dimensional subspace. In PCA, this projection consists of orthogonal components that maximize the variance of the data projected into the low-dimensional subspace [11]. In ICA, the projection consists of components that are as spatially independent as possible [12]. Each of these components is then assembled into brain maps with the value in each voxel representing the relative amount of that particular voxel, which is modulated by the activation of that component. Compared to the seed-based approach, both PCA and ICA have the advantage of providing automated components with no need for the pre-specification of a seed region, i.e., these methods are data-driven. The authors in reference [13] used ICA to decompose brain networks into spatial sub-networks with similar functions in both the resting state and task fMRI data.

Other methods combine the brain parcellation strategy used in seed-based methods with dimension reduction approaches to characterize brain circuitry. Reference [10] uses an anatomical atlas to pre-determine clusters (ROIs) and then extract features from each cluster via principal components. Multiple extracted components were then used to estimate connectivity between these ROIs using the RV coefficient, a measure that summarizes the correlation among sets of features.

In addition to the methods utilized to estimate connectivity, it is common to characterize the functional networks by using the tools of *graph theory*. In a graph, brain networks are treated as a collection of nodes connected by edges. Commonly, the edges are defined by an estimated connectivity. Following the specification of the nodes, a binary matrix is obtained by thresholding the connectivity matrix. The binary graph is then used to compute various graph parameters that describe the nature of the brain network. These parameters express key characteristics of the network and usually include quantities that help determine if the graph nodes are connected in a random or small-world order. Random networks have a more globally connected pattern while small-world networks show a high level of local ordering [14]. Statistical network models take these graph measures as inputs for the prediction of global networks that characterize multiple individuals.

The goal of this paper is to provide an overview of the most commonly used methods to estimate and characterize functional connectivity in resting-state fMRI data. We illustrate these methods by analyzing data from a single-subject in the Human Connectome Project. Although we do not attempt to offer an exhaustive presentation of the rapidly evolving methods in the field, we expect that the information provided here will guide researchers that are new to the field of neuroimaging in exploring these data.

The remainder of the paper is organized as follows: In Section 2, we describe the different methods of estimating functional connectivity, focusing on data from a single subject. In Section 3, we estimate functional connectivity for a single-subject resting-state data from the Human Connectome Project, using the methods described in Section 2. In Section 4, we present a few multiple-subject estimation methods. In Section 5, we describe statistical network models. Finally, in Section 6, we present some final remarks on the topic.

## 2. Methods for Functional Connectivity

In this section, we review the different methods to estimate functional connectivity for single-subject data. We focus on data for a single subject and discuss group connectivity in Section 4. For all calculations, let the matrix ***Y*** be a matrix of size $T\times N_{v}$, consisting of $N_{v}$ time courses representing the BOLD signal at each voxel $v=1,\ldots ,N_{v}$ [2] for a single subject. Here, for simplicity, we centralized the matrix ***Y*** by subtracting each voxel data (column) by the average of its corresponding time course. The goal of a connectivity-based analysis is to describe how various brain regions interact, either when the brain is resting or performing a task [15].

### 2.1. Seed-Based Analysis

It is computationally expensive to compute all pairwise correlations among a large number of voxels as it would require $N_{v}^{2}$ operations. Seed-based analysis (SBA) relies on estimating pairwise correlations between regions of interest (ROIs) or between a seed region and all the other voxels across the brain.

To estimate correlations between ROIs, a common approach is to divide the brain volume according to anatomical templates, usually called *brain atlases* [16]. There are several human brain atlases available, including the popular *Automated Anatomical Labelling (AAL)*, *Tailarach Atlas*, and the *MN1-152 atlas* [16,17]. To estimate correlations between a seed region and the other voxels, a seed is usually selected either by expert opinion or by choosing the voxel that shows greater activation during the fMRI experiment as the seed. The latter is more common during experiments involving tasks. After selection, the connectivity is estimated through a measure that quantifies correlation. Various measures were proposed in the literature. We provide more details about these measures in the Appendix A. We can summarize the seed-based approach the following way.

(i) Choose a seed region or voxel;
(ii) Correlate the time series of the region or voxel with all other voxels in the brain. If the seed is a region, average the time series of the region prior to correlating that with all other voxels in the brain. Use one of the measures described in Appendix A;
(iii) Display the 3D volumes of the correlation measure or display the thresholded correlations (just the ones that are significant). **Note:** To determine significance, we need to account for multiple comparisons. Bonferroni and FDR are widely used procedures.

Alternatively, after dividing the brain into various ROIs using an atlas, we can summarize the time series of that region, either by averaging across voxels or by calculating the first principal component [15]. Next, we use those summary time series to be correlated between all regions. We illustrate both options in Section 3.

### 2.2. Decomposition Methods

Although seed-based methods have a straightforward interpretation, they are biased to the seed selection technique [18] and, therefore, should be used with caution. Principal component analysis and independent component analysis aim at solving the issue by providing a data-driven approach to functional connectivity. These decomposition methods play many roles in functional neuroimaging applications. They are used in the pre-processing steps to remove data artifacts and to reduce data dimensionality, and they will likely appear in at least one step of estimating functional connectivity in various populations. In this section, we will focus on their role as a method to describe functional connectivity in single-subject fMRI data, while in Section 4, we explore their contribution in multi-subject analysis.

As an alternative to seed-based analysis, the goal of the decomposition methods is to represent the voxel domain as a smaller subset of spatial components. Each spatial component has a separate time course and represents simultaneous changes in the fMRI signals of many voxels [12]. In this section, we assume that for each column of ***Y*** the average was subtracted from the data.

#### 2.2.1. Principal Component Analysis (PCA)

PCA is a common method to reduce data dimensionality while minimizing the loss of data information and maximizing data variability [11]. The principal components are obtained either by the eigendecomposition of the sample covariance matrix $Y^{T}Y$ or by finding the eigenvectors of the data matrix ***Y*** using the theory of singular value decomposition (SVD). The rank of the data matrix is $r=min\left\{T,N_{v}\right\}$ (usually $T<N_{v}$ and r=T) and therefore we can find *r* principal components through the decomposition

(1) $$Y=U\Sigma W^{T}=\sum_{k=1}^{r}\sigma_{k}u_{k}w_{k}^{T},$$

where the T×r matrix ***U*** contains an orthonormal left singular vector $u_{k}\in {ℜ}^{T}$, the $r\times N_{v}$ matrix ***W*** contains orthonormal right singular vectors $w_{k}\in {ℜ}^{N_{v}}$, and the r×r diagonal matrix Σ contains the ordered singular values [11,15,19]. Notice that the eigendecomposition of $Y^{T}Y$ is defined as $W^{T}\Sigma^{2}W$. The orthonormal rows of the $r\times N_{v}$ matrix ***W*** are referred to as eigenimages and can be assembled into brain maps, each representing the relative amount from a given voxel that is modulated by the activation of that component.

A different approach is to project the original fMRI data into the space spanned by the first *p* principal components, where the choice of *p* is based on the amount of data variability explained by the component. The projected data matrix, YW, consists of the time series of regions in this new subspace. The authors in reference [20] used this idea to reduce the dimensionality of the fMRI data in certain ROIs and then applied a Granger causality analysis on the block time series of two brain regions to infer directional connections. Although it is possible to compute correlations using the time series of these projected data points, the results have no clear interpretation since each of these spatial regions in the new subspace represent a linear combination of different voxels in the original data space.

#### 2.2.2. Independent Component Analysis (ICA)

ICA aims at representing the brain data using a latent representation of independent factors. Differently from PCA, the goal is to decompose ***Y*** as a product of a mixing matrix and a combination of spatially independent components (ICs).

(2) $$Y=MC+E=\sum_{k=1}^{K}m_{k}c_{k}+E,$$

where ***M*** is the T×K mixing matrix with columns $m_{k}$, and the $K\times N_{v}$ matrix ***C*** is the matrix of independent components with rows $c_{k}$, where each $c_{k}$ contains brain networks corresponding to component *k* for a total of *K* independent components. These components represent the networks of various functions, such as motor, vision, auditory, etc. The elements of the matrix ***E*** are independent Gaussian noises.

It is assumed that the component maps, $c_{k},k=1,\ldots ,K$ represent possible overlapping and statistically dependent signals, but the individual component map distributions are independent, i.e., if $P(c_{k})$ represents the probability distribution of the voxels values in the *k*th component map, we have

(3) $$P\left(c_{1},c_{2},\ldots ,c_{K}\right)=\prod_{k=1}^{K}P\left(c_{k}\right).$$

Each independent component $c_{k}$ is a vector of size $N_{v}$ and can be assembled into brain maps. As in PCA, these maps represent the relative amount of a given voxel that is modulated by the activation of that component.

### 2.3. Computational Aspects

In imaging applications, estimating the principal components requires the decomposition of the $N_{v}\times N_{v}$ matrix $Y^{T}Y$, which is usually unfeasible. Many algorithms were proposed in the literature to efficiently estimate the components in such high-dimensional settings. Ref. [21] develops the sparse PCA (SPCA), which is based on a regression optimization problem using a lasso penalty, [22] a multilevel functional principal component for high-dimensional settings, and [23] estimate a sparse set of principal components through an iterative thresholding algorithm. Some of these toolboxes are easy to access and available for downloading at the authors’ website.

Similarly, estimating the independent components is not straightforward, and ranking the components is challenging because the ICs are usually not orthogonal, and the sum of the variances explained by each component will not sum to the variance of the original data. One of the first algorithms was the Infomax, which aims at maximizing the joint entropy of suitably transformed component maps [12,24]. Recently, more modern algorithms focus on extracting a sparse set of features from data matrices containing a very large number of features. Examples are the ICA with a reconstruction cost (RICA) proposed by [25], which is available as a Matlab toolbox.

### 2.4. A Hybrid Method

A different approach to estimate functional connectivity is given by reference [10]. The authors propose a multi-scale model based on networks at multiple topological scales, from voxel level to regions consisting of clusters of voxels, and larger networks consisting of collections of those regions. In practice, these collections of voxels are pre-specified and usually taken as anatomical ROIs. These anatomical ROIs can be then combined to form clusters of ROIs. Their approach consists of a dimension reduction step through to a factor model within each ROI. Let *r* represent the *r*-th ROI and $Y_{r}$ be a $T\times p_{r}$ data matrix consisting of the time series of voxels belonging to the *r*-th ROI (containing a total of $p_{r}$ voxels, where $\sum_{r=1}^{R}p_{r}=N_{v}$ and *R* is the total number of ROIs). Then, we write

(4) $$Y_{r}\left(t\right)=Q_{r}f_{r}\left(t\right)+E_{r}\left(t\right),$$

where $Y_{r}\left(t\right)$ is a column vector of size $p_{r}$, $f_{r}\left(t\right)$ is a $m_{r}\times 1$ vector of latent common factors with a number of factors $m_{r}\ll p_{r}$, $Q_{r}$ is a $p_{r}\times m_{r}$ factor-loading matrix that defines the dependence between the $p_{r}$ voxels through the mixing of $f_{r}$, and $E_{r}\left(t\right)={\left[e_{r1}\left(t\right),\ldots ,e_{rp_{r}}\left(t\right)\right]}^{\prime }$ is a $p_{r}\times 1$ vector of white noise with $E(E_{r}\left(t\right))=0$ and $\Sigma_{E_{r},E_{r}}=Cov\left(E_{r}\left(t\right)\right)=\mathrm{diag}\left(\sigma_{e_{r1}}^{2},\ldots ,\sigma_{e_{rp_{r}}}^{2}\right)$.

These factor models are then concatenated to define

(5) Y(t)=Qf(t)+E(t),

where Y(t) is a column vector of size $\sum_{r=1}^{R}p_{r}=N_{v}$, $Q=\mathrm{diag}(Q_{1},\ldots ,Q_{R})$ is a $\sum_{r=1}^{R}p_{r}\times \sum_{r=1}^{R}m_{r}$ block-diagonal mixing matrix and $f\left(t\right)={\left[f_{r}\left(t\right),\ldots ,f_{R}\left(t\right)\right]}^{\prime }$ is a $\sum_{r=1}^{R}m_{r}\times 1$ vector of aggregated latent factors.

Network covariance matrices in these different topological scales are estimated using the low-rank matrix in the following way. Let $\Sigma_{Y_{r}Y_{r}}$ be the covariance matrix within ROI *r*. Model (4) implies the following decomposition

(6) $$\Sigma_{Y_{r}Y_{r}}=Q_{r}\Sigma_{f_{r}f_{r}}Q_{r}^{\prime }+\Sigma_{E_{r}E_{r}}.$$

Similarly, from Model (5) we have

(7) $$\Sigma_{YY}=Q\Sigma_{ff}Q^{\prime }+\Sigma_{EE}.$$

The low-dimensional factor covariance matrix $\Sigma_{ff}$ is a block matrix used to estimate the lag-zero dependency between ROIs as follows.

$$\Sigma_{ff}=\left(\begin{array}{ccc} \Sigma_{f_{1}f_{1}} & & \Sigma_{f_{1}f_{R}}\\ & \ddots & \\ \Sigma_{f_{R}f_{1}} & & \Sigma_{f_{R}f_{R}} \end{array}\right)$$

The diagonal blocks $\Sigma_{f_{r}f_{r}},r=1,\ldots ,R$ are diagonal covariance matrices, capturing the total variance of factors within each ROI. The off-diagonal blocks $\Sigma_{f_{k}f_{j}},j\neq j$ are cross-covariance matrices between factors and summarize the dependence between clusters j and k.

The authors summarize the dependence between ROIs using the RV coefficient, a multivariate generalization of the squared correlation coefficient. The RV coefficient between factors in clusters j and k is defined by

(8) $$RV=\frac{\mathrm{tr}(C_{f_{k}f_{j}},C_{f_{j}f_{k}})}{\sqrt{\mathrm{tr}\left(C_{j_{k}f_{j}},C_{f_{j}f_{j}}\right)\mathrm{tr}\left(C_{f_{k}f_{k}},C_{f_{k}f_{k}}\right)}},$$

where $C_{f_{j}f_{k}}={\left(\Sigma_{f_{j}f_{j}}\right)}^{-\frac{1}{2}}\Sigma_{f_{j}f_{k}}{\left(\Sigma_{f_{k}f_{k}}\right)}^{-\frac{1}{2}}$.

In practice, the authors apply this model to estimate resting-state networks. They estimate the factors $f_{r}$ and matrices $Q_{r}$ using PCA and pre-specify the ROIs based on an anatomical atlas. The authors in reference [26] use this approach to estimate background functional connectivity between ROIs using data from the Working Memory Task in the Human Connectome Project.

### 2.5. Brain Networks

It is common to represent the brain using tools from *graph theory*. In this framework, we can think of functional connectivity as a network represented by a graph, where the spatial units are the nodes and the connection between them are the edges. Networks are treated as a collection of nodes (vertices) connected by links (edges). The graph (network) is represented as the pair G=(V,E), where *V* and *E* are the sets of vertices and edges, respectively. In addition, graphs may be weighted and, in such cases, will be denoted by the triple G=(V,E,W), with W(E) indicating the weight for each edge.

The first decision to make is the selection of the nodes of the network. Similar to the seed-based connectivity, these nodes are defined by either voxels or the ROI parcellations given by anatomical atlases. Following the specification of the nodes, their edges (links) must be determined. These edges quantify the strength of association between these different nodes, i.e., they are the functional connectivity. The same measures discussed previously for functional connectivity and described in Appendix A are used to quantify the strength of the edges.

Most of the standard tools of graph theory have been developed for binary networks, where each edge is either present or not. A binary matrix, usually called an *adjacency matrix*, is obtained by thresholding the connectivity matrix. Although it is convenient to threshold the weighted graphs to apply the standard graph theoretical machinery, information about the original signal is discarded in the process. Moreover, in most situations, the choice of a threshold is not unique, and such a decision may be difficult to justify. One strategy is the use of a mass-univariate approach, in which a statistical test is performed for every possible edge in the network and then corrected for multiple comparisons using standard techniques, such as the Bonferroni correction or the false discovery rate (FDR) [27,28].

After the network is estimated, some descriptive measures are calculated as means to describe the topological graph properties. In brain networks, the popular metrics are the characteristic path length, the clustering coefficient, and the degree distribution. For a list of the comprehensive topological measures used in neuroimaging, see reference [29].

**Characteristic path length.** Paths are the sequences of distinct nodes that represent the potential flow of information between pairs of brain regions with shorter paths, implying stronger potential for integration. The length of a path estimates the potential for functional integration between brain regions. One of the most commonly used measures of functional integration is the average shortest path length between all pairs of nodes in the network, which is defined as the characteristic path length [15]. Paths between disconnected nodes are defined to have infinite length, which is a problem when calculating this measure, especially in sparse networks such as in functional connectivity. In practice, we take the average only between the existing paths, which can be a problem. For a discussion on this issue please refer to reference [29].
**Degree distribution.** A measure of centrality, the degree of an individual node is equal to the number of links connected to that node, i.e., the number of neighbors of the node. The degree distribution is, therefore, the distribution of the degrees of all the nodes in the network. In functional connectivity, nodes with a high degree are interacting functionally with many other nodes in the network [29] and are referred to as *hubs*.
**Clustering coefficient.** A measure of segregation, the clustering coefficient is the fraction of the node’s neighbors that are also neighbors of each other, which in graph theory is the fraction of triangles around an individual node. The presence of clusters in functional networks suggests an organization of statistical dependencies indicative of segregated functional neural processing, which is the ability for specialized processing to occur within densely interconnected groups of brain regions. The mean clustering coefficient for the network reflects, on average, the prevalence of clustered connectivity around individual nodes. The mean clustering coefficient is normalized individually for each node and can disproportionately be influenced by nodes with a low degree.

Many other network measures are greatly influenced by basic network characteristics, such as the number of nodes and links and the degree of distribution presented in this section.

## 3. Real Data Example

We analyzed the resting-state data from the Human Connectome Project (HCP). We chose to work with the data that had been previously denoised using the FIX pipeline [30]. This pipeline uses a gentle high-pass temporal filter, performs motion regression (i.e., the regression of 24 movement parameters: six rigid-body motion parameters, their backward temporal derivatives, and squares of those 12 time series), and applies a regression based on ICA to remove the variance in noise components that was orthogonal to signal components [31]. For the single-subject analysis, we considered the volumes collected from the right–left phase of the example, Subject 100307. Volumes of fMRI were obtained every 720 ms. Each volume consisted of images of size 91×109×91 for a total of 1200 time frames.

### 3.1. Single-Subject Examples

#### 3.1.1. ROI-Based Connectivity

We partitioned the brain into ROIs using the AAL Atlas version that was registered into the MNI152 space. We considered a total of 166 ROIs and estimated the connectivity using the following methods:

(a) Cross correlation of the average time series in each ROI;
(b) Partial correlation of the average time series in each ROI;
(c) Cross correlation of the time series of the ROI data projected into the space of its first principal component;
(d) Partial correlation of the time series of the ROI data projected into the space of its first principal component;
(e) For each ROI, we consider the principal components that account for 20% of the ROI variability and calculate the RV coefficient as described in Equation (8).

**Figure 1:**
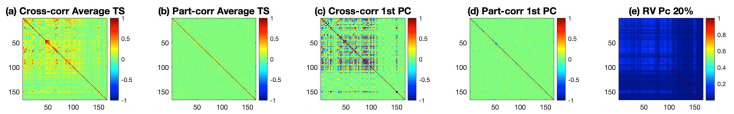
Estimated connectivity for the ROIs based on the AAL parcellation. Panel (**a**) depicts the cross-correlation for the average time series of the ROIs, panel (**b**) depicts the partial cross correlation for the average time series of the ROIs, panel (**c**) depicts the cross correlation for the time series of the ROI data projected with the first PC, panel (**d**) depicts the partial cross correlation for the time series of the ROI data projected with the first PC, and panel (**e**) represents the RV coefficient with each ROI retaining the principal components that explain 20% of its variability.

The results for the estimated connectivity values are shown in Figure 1. Inspecting Figure 1 reveals that cross-correlation measures in panels (a) and (c) capture larger correlations than their corresponding partial cross-correlation measures (panels (b) and (d)). The RV coefficient from the method described in (e) seems to be able to capture a large number of small correlations among ROIs. Before drawing any conclusions from the figure, we should first test whether these values are significant. For the first four matrices, the test is done by first transforming these values to z-scores and then thresholding them to identify important correlations. For the RV coefficient in panel (e), significance is based on the asymptotic distribution of the coefficient as detailed in reference [10].

**Figure 2:**
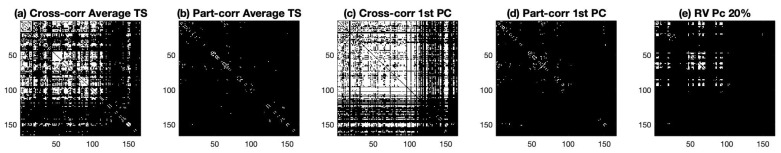
Binary Graphs obtained from the thresholded connectivities matrices of Figure 1. For all panels, the white color indicates an edge between the ROIs. Panel (**a**) is the graph obtained by thresholding the cross correlation of the average time series of the ROIs, panel (**b**) depicts the graph from the thresholded partial cross correlation for the average time series of the ROIs, panel (**c**) depicts the graph obtained by thresholding the cross correlation for the time series of the ROI data projected with the first PC, panel (**d**) depicts the graph obtained by thresholding the partial cross correlation for the time series of the ROI data projected with the first PC, and panel (**e**) represents the graph obtained by thresholding the RV coefficient.

Next, we used these connectivity matrices to obtain a binary graph with the edges determined based on the *p*-values obtained from the z-scores of the correlation coefficient, as described in Appendix A, Equation (A2). The *p*-values were thresholded based on the Bonferroni correction and a significance of 5%. For the RV coefficient in panel (e), we use the asymptotic distribution of the coefficients to convert the values to z-scores and thresholded based on the Bonferroni correction to find the quantile of the standard normal distribution with a significance of 5%. Considering this criteria, we compute the adjacency matrices shown in Figure 2.

Inspecting Figure 2 reveals the presence of a large number of edges for both (a) and (c) graphs. This indicates a high level of interaction between the different anatomical regions. This high-interaction level was not found in graphs (b) and (d). In panel (e), we observe a moderate level of interaction with a few ROIs connecting with many others, while some regions are quiet during the resting-state experiment.

#### 3.1.2. Network Summary Measures

We used the binary graphs obtained above to estimate a few summary measures, using graph theory as described in Section 2.5. The formulas used in each calculation are detailed in Appendix B. Table 1 shows the results. *CPL* is the characteristic path length excluding all infinity paths from the network, *DG* is the average degree of the network, where the degree indicates the number of links in each node, *CC* is the average clustering coefficient of the network, and *Inf* is the number of infinity paths in the network. The quantities in Table 1 reflect what we observe in Figure 2. The degree indicates the number of connections between regions. As noticed before, the graphs in panels (a) and (c) indicate a high degree, with many interactions between ROIs. The characteristic path length (CPL) of the RV coefficient indicates that on average the network has a short path length, with a value that is comparable to the networks in panels (a) and (c) of Figure 2. This indicates that despite few regions being connected, the ones that are connected are near each other.

#### 3.1.3. Volume-Based Connectivity

**Figure 3:**
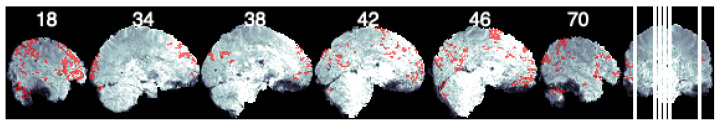
Seed- based connectivity of the left pars opercularis. Figure shows sagittal slices with voxels that have a significant connection with the seed ROI depicted in red.

**Seed-Based Analysis.** For seed-based analysis, we chose the left pars opercularis (left interior frontal gyrus) as the seed [32]. We take the average time series for this region and compute the cross correlation with the remaining voxels. We perform a Bonferroni correction considering α=0.05 to threshold the correlation values. Figure 3 shows the resulting brain map. We display clusters bigger than 125 as significant voxels, and their mask is overlaid on a template brain consisting of the average time points of the example subject data used here.

**Figure 4:**
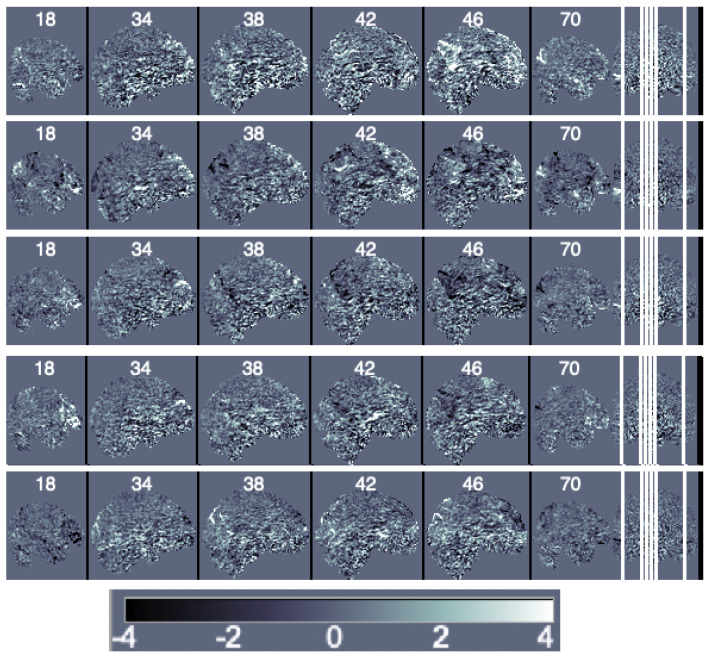
Sagittal view of the ordered principal components’ maps from first (**top**) to fifth (**bottom**).

**Decomposition Methods.** We first obtain the principal components of the data matrix ***Y***. It is important to notice that a large number of principal components is needed to represent data variability and that traditional principal components have the issues discussed in Section 2.3. For this particular data, 150 components are needed to represent 20% of the data variability and 463 are needed to represent 50%. We illustrate the first five components scaled by their eigenvalues (i.e., the loadings) in Figure 4.

**Figure 5:**
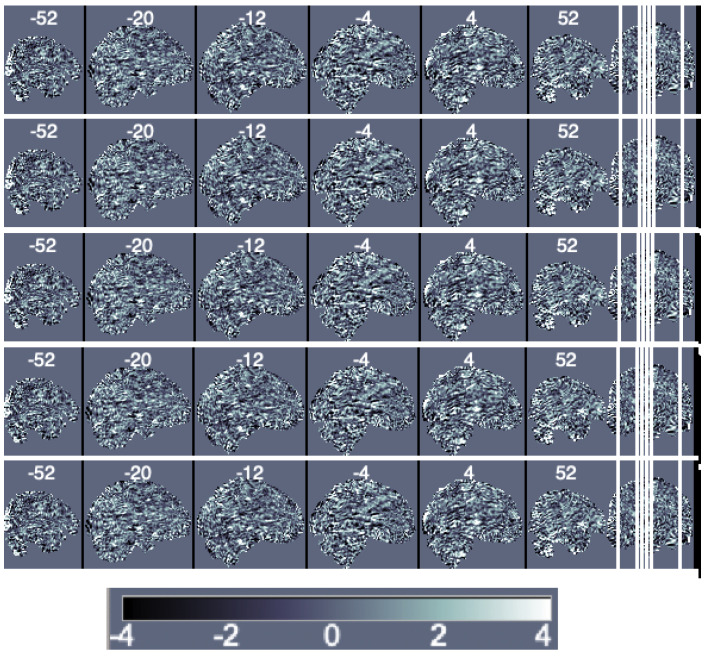
Sagittal view of the independent components’ maps ordered based on increasing amounts of uniquely explained variance from first (**top**) to fifth (**bottom**).

Next, to estimate the independent components, we use the probabilistic independent components analysis proposed in reference [33] and implemented in the MELODIC (multivariate exploratory linear optimized decomposition into independent components) function in FSL. Figure 5 depicts the results.

For illustration purposes, we present the components without thresholding their values. It is more common to use the individual components’ maps as inputs in a multi-subject approach and then perform thresholding in the group components to identify a group network. We comment more on the topic in the next section.

## 4. Multiple-Subject Functional Connectivity

When modeling functional MRI, an important goal is to identify the functional connectivity structure in multi-subject data by leveraging a shared structure across subjects. Multi-subject functional connectivity models can range from constrained tensor decomposition models, e.g., PARAFAC, to more flexible approaches where subject-specific connectivity matrices or PCA and ICA models are estimated first, and their concatenated results are used as inputs on a group-based estimation. The optimal model will depend on which level of flexibility best captures the functional connectivity features within the group [34].

In multi-subject ICA models, a simple procedure is to estimate the single-subject connectivity matrix using pre-specified ROIs, as in the seed-based approach described in Section 2, and then aggregate those results into a single matrix, subsequently further decomposing this matrix using principal components. The principal components can then be mapped to estimate a group-based functional connectivity. Ref. [35] used this idea to estimate a dynamical group-based resting-state connectivity of minimally disabled relapsing–remitting patients.

A multi-stage approach is implemented in reference [36] to compare functional connectivity between subjects at a high familial risk for Alzheimer’s disease that are clinically asymptomatic versus matched controls. The method follows four steps, including subject-specific SVD, a population-level decomposition of aggregated subject-specific eigenvectors, a projection of the subject-level data onto the population eigenvectors to obtain subject-specific loadings, and the use of the subject-specific loadings in a functional logistic regression model.

A group of methods propose a *group ICA* approach, where fMRI data is either temporally concatenated across subjects or taken as a multi-dimensional array. The FMRIB Software Library (FSL), a software library containing image analysis and statistical tools for various imaging data, provides group ICA and tensorial ICA in its MELODIC function.This section will focus on these two approaches.

### 4.1. Group ICA

Ref. [37] proposed for the first time an approach to perform ICA on fMRI data from a group of subjects. Suppose we observe fMRI data from *n* subjects. Let $Y_{i}$ be a matrix of size $T\times N_{v}$ consisting of $N_{v}$ time courses representing the BOLD signal at each voxel $v=1,\ldots ,N_{v}$ for subject i=1,…,n. Their model involves a multi-stage approach as follows.

1. Subject-level data reduction. In this step, reduction is applied in the temporal domain. For each subject i=1,….n, the reduced data is given by
  $$X_{i}=F_{i}^{-1}Y_{i},$$
  where $F_{i}^{-1}$ is a L×T reducing matrix and $X_{i}$ is a $L\times N_{v}$ matrix representing the reduced data. In practice, $F^{-1}$ is obtained by PCA decomposition;
2. Data reduction of the aggregated subject-level data. Data reduction is applied to the $NL\times N_{v}$ matrix ${\left[X_{1}^{T},\ldots ,X_{N}^{T}\right]}^{T}$ to obtain a $K\times N_{v}$ matrix $X=G^{-1}{\left[X_{1}^{T},\ldots ,X_{N}^{T}\right]}^{T}$, where *K* is the number of components to be obtained and $G^{-1}$ is a K×NL-reducing matrix that is in practice obtained by principal components;
3. Estimation of independent sources. An ICA decomposition is applied to the matrix ***X***, as described in Section 2.2.2.
  X=MC,
  where ***M*** is a K×K-mixing matrix and ***C*** is a $K\times N_{v}$ component map matrix. The resulting group ICA components can be thresholded by first converting them into Z-scores.

Individual maps can be obtained by partitioning GM (where $G={\left(G^{-1}\right)}^{T}$) by subject and going back along the previous steps as follows.

$$GX=GMC=\left[\begin{array}{c} F_{1}^{-1}Y_{1}\\ \ddots \\ F_{N}^{-1}Y_{N} \end{array}\right].$$

Based on these steps, the matrix GMC is a matrix of size $NL\times N_{v}$ of individual maps and can be partitioned such that $G_{i}M_{i}C_{i}=F_{i}^{-1}Y_{i}$, and $C_{i}$ contains the individual maps.

### 4.2. Tensorial ICA

The tensor ICA is based on tensor decomposition, which obtains a low-rank representation of a multi-dimensional array. PARAFAC is a common decomposition method [38]. Let $X\in R^{T\times N_{v}\times N}$ be an array with fMRI data and dimension times, voxels, and subjects, respectively. The three-dimensional array ***X*** can be decomposed as a sum of *R* outer products in the following way

$$X=\sum_{r=1}^{R}a_{r}\circ b_{r}\circ c_{r},$$

where $a_{r}\in R^{T}$, $B_{r}\in R^{N_{V}}$, and $c_{r}\in R^{N}$. This decomposition implies that each element in the array ***X*** can be written as

$$\left\{x_{ijk}\right\}=\sum_{r=1}^{R}a_{ir}b_{jr}c_{kr}.$$

The vectors in the decomposition can be represented in matrices, e.g., $A=[a_{1}a_{2}\ldots a_{R}]$, and likewise to obtain matrices ***B*** and ***C***. The three-dimensional array can be unfolded into matrices in a process called matricization. The unfolding can happen in any of the three dimensions. On the second dimension, $X_{\left(2\right)}\in R^{N_{v}\times NT}$ is the mode-two matricization of ***X***. Similarly, we can use the unfolding to generate mode-two and mode-three matrices. For details on the PARAFAC decomposition and matricization, please refer to reference [38]. Using these definitions, we can write:

$$X_{\left(2\right)}=B{\left(C⊙A\right)}^{T},$$

where ⊙ denotes the Katri–Rao product. In reference [39], the authors propose an ICA decomposition of the form

$$X^{*}=\left(C⊙A\right)B^{T}+E,$$

where $X^{*}=X_{\left(2\right)}^{T}$ and the mixing matrix M=(C⊙A) and component matrix $B^{T}$ are estimated as in reference [33].

## 5. Statistical Network Models

In this section, we follow the notation in reference [40] to describe statistical network models with the purpose of characterizing brain circuitry. In these models, individual functional connectivity is estimated first, using the techniques described in Section 2. After individual estimation, the effects of multiple variables of interest and topological network features are taken into account on the overall network structure.

Let $\left(Y_{i},X_{i}\right)$ represent the network and covariates for subject *i*, respectively. The probability density function of the network given the covariates is denoted by $P(Y_{i}|X_{i},\theta_{i})$, where $\theta_{i}$ describes the relationship of $Y_{i}$ and $X_{i}$. These covariates can be node-specific covariates, such as brain location and also functions of the network $Y_{i}$, such as the path length or other metrics described in Section 2.5. Popular ways of modeling the density function include exponential random graph models (ERGMs) and mixed models [40].

In ERGMs, we consider binary graphs and the models are fitted for each subject individually as follows. Let $Y_{i}$ be a network consisting of R×R nodes. Then, $Y_{ijk}=1$ if a link exists between nodes *j* and *k*, and $Y_{ijk}=0$ otherwise. The probability mass function has the form of a regular exponential family:

$$P\left(Y_{i}=y_{i}|X_{i}\right)=\kappa {\left(\theta \right)}^{-1}exp\left\{\theta^{T}g\left(y_{i},X_{i}\right)\right\}.$$

The estimation of the parameters θ is done by MCMC MLE. In reference [41], they identify the most important explanatory metrics $g(y_{i})$ for each subject’s network. Next, the authors create a group-based summary measure of the fitted parameter values θ for all subjects. They use these group-based explanatory metrics and parameters to fit a group-based representative network via ERGMs.

One limitation of the current estimation methods for ERGMs is scalability. The major issue is not the number of ROIs per se but the edge structure of the network, which can cause convergence problems. Moreover, most models were developed for binary graphs and are not well-suited for link-level examination [40].

As an alternative to ERGMs, mixed models allow for both link-level examination and multiple-subject comparisons. The framework defines a two-part mixed effect that models both the probability of a connection being present or absent and the strength of a connection if it exists. Let $Y_{i}$ be a representation of the functional connectivity strength given by one of the correlation measures listed in Appendix A, and let $R_{ijk}$ be an indicator of whether a connection between *j* and *k* is present. Then the conditional probabilities are

$$P\left(R_{ijk}=r_{ijk}|\beta_{r};b_{ri}\right)=\left\{\begin{array}{cc} 1-p_{ijk}\left(\beta_{r};b_{ri}\right), & \mathrm{if}\;r_{ijk}=0\\ p_{ijk}\left(\beta_{r};b_{ri}\right), & \mathrm{if}\;r_{ijk}=1, \end{array}\right.$$

where $\beta_{r}$ are the vector of fixed effects that relate the covariates $X_{ijk}$ for each participant and pair of nodes, and $b_{ri}$ are random effects representing subject-specific and node-specific parameters.

Let $Z_{ijk}$ be the design matrix associated with the random effects $b_{ri}$; the models are divided into two parts. The first part of the model uses a logit link function to relate the probability of connection between nodes *j* and *k* to the covariates as follows.

$$\mathrm{logit}\left(p_{ijk}\right)=X_{ijk}^{\prime }\beta_{r}+Z_{ijk}^{\prime }b_{ri}.$$

The second part models the strength of the connection between nodes *j* and *k* given that the connection is present, by linearly linking the Fisher’s Z-transform of the correlation coefficient between nodes *i* and *j* and the covariates. Let $S_{ijk}=Y_{ijk}|R_{ijk}=1$, then

$${\mathrm{Fisher}}^{\prime }s\;Z-\mathrm{transform}\left(S_{ijk}\right)=X_{ijk}^{\prime }\beta_{s}+Z_{ijk}^{\prime }b_{si}+e_{ijk},$$

where $\beta_{r}$ is a vector of population parameters that related the strength of connection to the same set of covariates $X_{ijk}$ for each participant and pair of nodes, $b_{si}$ is a vector of subject and node-specific parameters that capture how this relationship varies about the population average $\beta_{s}$, and $e_{ijk}$ is the random noise for subject *i* and nodes *j* and *k*. Details of the two-parts modeling approach is presented in reference [42].

One issue that arises from these models is that thresholding choices based on the connectivity weights will impact the network topology, even if multiple comparisons are taken into account. The authors in reference [40] argue that persistent homology provides a multi-scale hierarchical framework that addresses the threshold issue. The method is a technique of computational topology that provides a coherent mathematical framework for comparing networks. Instead of looking at the networks at a fixed threshold, persistent homology records the changes in topological network features over multiple resolutions and scales. By doing so, it reveals the features that are robust to noise, i.e., the most ‘persistent’ topological features.

## 6. Summary

In this paper, we have reviewed the most common methods to estimate functional connectivity in fMRI data. For single-subject data, estimation can be done by directly quantifying correlations across regions of interest and/or seed regions, or by finding a set of latent components that represent simultaneous activity, and while interpretation is straightforward for the former approach, it is not as clear for the later. In the example provided, the number of component maps needed to represent the data variability is very high and, therefore, the investigation of only a few components might not reflect the whole picture of the brain network.

The results obtained in Section 2 indicate that even if the regions are defined in an equivalent way, different estimation procedures of connectivity will lead to different interpretations of the networks. Therefore, it is of great importance to be aware of the limitations of each approach, especially when interpreting results from individual datum.

Despite the challenges with the single-subject analysis, a consistent procedure, applied to various subjects, might translate into a successful representation of multiple-subject networks. This is specially true if the method does not require a multi-stage approach and performs, instead, a joint estimation as in the tensorial ICA framework. Other emerging multi-subject network methods, such as persistent homology, are a promising way to estimate brain circuitry, especially if scalability can be achieved.

## Figures and Tables

**Table 1:** Network summary measures.

	(a) Av. CCorr	(b) Av. Pcorr	(c) PC1 Ccorr	(d) PC1 Pcorr	(e) RV
CPL	2.137	5.029	1.508	3.9685	1.8789
DG	34.193	1.313	69.518	1.386	4.217
CC	0.640	0.072	0.825	0.179	0.820
Inf	2963	11,239	2964	11,789	12,366

## Appendix A. Methods to Quantify Correlation

**Cross Correlation**. Cross correlation measures the (lagged) temporal dependencies between two signals, and it was first proposed by reference [43] as an effective way to describe functional connectivity. Suppose we want to calculate the correlation between the BOLD time series for a given voxel *v*, i.e., $y_{v}\left(t\right),t=1,\ldots ,T$ and a reference time series $r_{v^{\prime }}\left(t\right),t=1,\ldots ,T$ for $v\neq v^{\prime }$. Let $\mu_{y}$ and $\mu_{r}$ be the average value of the vectors $y_{v}$ and $r_{v^{\prime }}$, respectively. Then, the cross correlation between the vectors $y_{v}$ and $r_{v^{\prime }}$ is defined as

(A1) $$cc_{y,r}=\frac{\sum_{t=1}^{T}\left(y_{v}\left(t\right)-\mu_{y}\right)\left(r_{v^{\prime }}\left(t\right)-\mu_{r}\right)}{\sqrt{\sum_{t=1}^{T}{\left(y_{v}\left(t\right)-\mu_{y}\right)}^{2}}\sqrt{\sum_{t=1}^{T}{\left(r_{v^{\prime }}\left(t\right)-\mu_{r}\right)}^{2}}}$$

The reference vector can be a pre-selected voxel, the seed, or it can be an average of time series in a certain region. For cross correlation between ROIs, both y(t),t=1,…,T and r(t),t=1,…,T can be the average time series in the pre-determined regions *y* and *r*, respectively.

It is common to transform the correlation coefficient obtained in (A1) using a Fisher’s Z-transformation for each correlation coefficient as follows

(A2) $$z-\mathrm{score}=\frac{ln\left(1+cc_{y,r}\right)-ln\left(1-cc_{y,r}\right)}{2}.$$

These coefficients are approximately normally distributed, and cutoff values are obtained from the standard normal distribution.

**Partial Cross Correlation.** Cross correlation quantifies only the marginal linear dependence between two signals and does not consider the effect of a third signal [15,44]. To remove the linear influence of a third signal k(t) we define the partial correlation as follows.

(A3) $$PCC_{y,r|k}=\frac{cc_{y,r}-cc_{y,k}cc_{r,k}}{\sqrt{1-cc_{y,k}^{2}}\sqrt{1-cc_{r,k}^{2}}}.$$

Partial cross correlation is a valuable metric for estimating brain networks because it can estimate the direct relationship between two signals [15].

The calculation of cross correlation and partial cross-correlation measures assumes the signals to be stationary. When this assumption is not satisfied, detrended cross correlation and detrended partial cross correlation should be used instead [45].

**Time-varying connectivity.** It is possible to obtain a dynamical functional connectivity to understand its pattern over time. Both static measures mentioned in this section have a natural time-varying analogue in conjunction with a sliding window [15].

The concept of the sliding window is simple. Starting from the first time point, a window (a fixed number of time points) is selected, and all data points within the window are used to estimate the FC. This window is then shifted a certain number of time points, and the FC is estimated on the new set of data points. The process is repeated until the end of the time course. The series of estimated FC over time is the time-varying FC.

## Appendix B. Calculation of Network Measures

For completeness, we present the mathematical definitions of the network measures presented in Section 2.5. For a complete list of network measures, please refer to reference [29].

We use the graph notation as defined in Section 2.5. Let *n* be the number of nodes in the network and *N* be the set of all nodes. Let *l* be the number of links in the network and *L* be the set of all links. Then, (i,j) is a link between nodes *i* and *j* and $a_{ij}=1$ when there is a link (i,j). We define $l=\sum_{i,j}a_{ij}$ (counting each indirect link twice).

**Degree of a node.** The degree of a node *i* is the sum of all the links connected to the node and is defined as
  $$k_{i}=\sum_{j\in N}a_{ij}.$$
**Shortest path length.** The shortest path length measures the shortest distance between nodes *i* and *j* and is defined as:
  $$d_{ij}=\sum_{a_{uv}\in g_{i↔j}}a_{uv},$$
  where $g_{i↔j}$ is the shortest distance between *i* and *j*. For all disconnected pairs (i,j), $d_{ij}=\infty$.
**Characteristic path length.** Let $L_{i}$ be the average distance between node *i* and all other nodes. The characteristics path length is defined as
  $$L=\frac{1}{n}\sum_{i\in N}L_{i}=\frac{1}{n}\sum_{i\in N}\frac{\sum_{j\in N,j\neq i}d_{ij}}{n-1}.$$
**Number of triangles.** The number of triangles of a node *i* is defined as
  $$t_{i}=\frac{1}{2}\sum_{j,h\in N}a_{ij}a_{ih}a_{jh}.$$
**Clustering coefficient.** The clustering coefficient of the network is defined as
  $$C=\frac{1}{n}\sum_{i\in N}C_{i}=\frac{1}{n}\sum_{i\in N}\frac{2t_{i}}{k_{i}\left(k_{i}-1\right)}.$$

## References

[1] Elam J.S., Glasser M.F., Harms M.P., Sotiropoulos S.N., Andersson J.L., Burgess G.C., Curtiss S.W., Oostenveld R., Larson-Prior L.J., Schoffelen J.M. (2021). The Human Connectome Project: A retrospective. NeuroImage.

[2] Ogawa S., Lee T.M., Kay A.R., Tank D.W. (1990). Brain magnetic resonance imaging with contrast dependent on blood oxygenation. Proc. Natl. Acad. Sci. USA.

[3] Barch D.M., Burgess G.C., Harms M.P., Petersen S.E., Schlaggar B.L., Corbetta M., Glasser M.F., Curtiss S., Dixit S., Feldt C. (2013). Function in the human connectome: Task-fMRI and individual differences in behavior. NeuroImage.

[4] Van den Heuvel M.P., Hulshoff Pol H.E. (2010). Exploring the brain network: A review on resting-state fMRI functional connectivity. Eur. Neuropsychopharmacol..

[5] Biswal B.B., Kylen J.V., Hyde J.S. (1997). Simultaneous assessment of flow and BOLD signals in resting-state functional connectivity maps. NMR Biomed..

[6] Belliveau J.W., Cohen M.S., Weisskoff R.M., Buchbinder B.R., Rosen B.R. (1991). Functional studies of the human brain using high-speed magnetic resonance imaging. J. Neuroimaging.

[7] Glover G.H. (2011). Overview of functional magnetic resonance imaging. Neurosurg. Clin..

[8] Wang L., Zang Y., He Y., Liang M., Zhang X., Tian L., Wu T., Jiang T., Li K. (2006). Changes in hippocampal connectivity in the early stages of Alzheimer’s disease: Evidence from resting state fMRI. NeuroImage.

[9] Rajamanickam K. (2020). A Mini Review on Different Methods of Functional-MRI Data Analysis. Arch. Intern. Med. Res..

[10] Ting C., Ombao H., Salleh S., Latif A.Z.A. (2020). Multi-Scale Factor Analysis of High-Dimensional Functional Connectivity in Brain Networks. IEEE Trans. Netw. Sci. Eng..

[11] Jolliffe I.T., Cadima J. (2016). Principal component analysis: A review and recent developments. Philos. Trans. R. Soc. A Math. Phys. Eng. Sci..

[12] Mckeown M.J., Makeig S., Brown G.G., Jung T.P., Kindermann S.S., Bell A.J., Sejnowski T.J. (1998). Analysis of fMRI data by blind separation into independent spatial components. Hum. Brain Mapp..

[13] Smith S.M., Fox P.T., Miller K.L., Glahn D.C., Fox P.M., Mackay C.E., Filippini N., Watkins K.E., Toro R., Laird A.R. (2009). Correspondence of the brain’s functional architecture during activation and rest. Proc. Natl. Acad. Sci. USA.

[14] Van den Heuvel M., Stam C., Boersma M., Hulshoff Pol H. (2008). Small-world and scale-free organization of voxel-based resting-state functional connectivity in the human brain. NeuroImage.

[15] Ombao H., Lindquist M., Thompson W., Aston J. (2016). Handbook of Neuroimaging Data Analysis.

[16] O’Reilly J.X., Woolrich M.W., Behrens T.E., Smith S.M., Johansen-Berg H. (2012). Tools of the trade: Psychophysiological interactions and functional connectivity. Soc. Cogn. Affect. Neurosci..

[17] Tzourio-Mazoyer N., Landeau B., Papathanassiou D., Crivello F., Etard O., Delcroix N., Mazoyer B., Joliot M. (2002). Automated Anatomical Labeling of Activations in SPM Using a Macroscopic Anatomical Parcellation of the MNI MRI Single-Subject Brain. NeuroImage.

[18] Wu L., Caprihan A., Bustillo J., Mayer A., Calhoun V. (2018). An approach to directly link ICA and seed-based functional connectivity: Application to schizophrenia. NeuroImage.

[19] Andersen A.H., Gash D.M., Avison M.J. (1999). Principal component analysis of the dynamic response measured by fMRI: A generalized linear systems framework. Magn. Reson. Imaging.

[20] Zhou Z., Ding M., Chen Y., Wright P., Lu Z., Liu Y. (2009). Detecting directional influence in fMRI connectivity analysis using PCA based Granger causality. Brain Res..

[21] Zou H., Hastie T., Tibshirani R. (2006). Sparse Principal Component Analysis. J. Comput. Graph. Stat..

[22] Zipunnikov V., Caffo B., Yousem D.M., Davatzikos C., Schwartz B.S., Crainiceanu C. (2011). Multilevel Functional Principal Component Analysis for High-Dimensional Data. J. Comput. Graph. Stat..

[23] Ma Z. (2013). Sparse principal component analysis and iterative thresholding. Ann. Stat..

[24] Bell A.J., Sejnowski T.J. (1995). An information-maximization approach to blind separation and blind deconvolution. Neural Comput..

[25] Le Q., Karpenko A., Ngiam J., Ng A., Shawe-Taylor J., Zemel R., Bartlett P., Pereira F., Weinberger K.Q. (2011). ICA with Reconstruction Cost for Efficient Overcomplete Feature Learning. Advances in Neural Information Processing Systems.

[26] Miranda M.F., Morris J.S. (2021). Novel Bayesian method for simultaneous detection of activation signatures and background connectivity for task fMRI data. arXiv.

[27] He Y., Chen Z.J., Evans A.C. (2007). Small-World Anatomical Networks in the Human Brain Revealed by Cortical Thickness from MRI. Cereb. Cortex.

[28] Achard S., Salvador R., Whitcher B., Suckling J., Bullmore E. (2006). A Resilient, Low-Frequency, Small-World Human Brain Functional Network with Highly Connected Association Cortical Hubs. J. Neurosci..

[29] Rubinov M., Sporns O. (2010). Complex network measures of brain connectivity: Uses and interpretations. NeuroImage.

[30] Salimi-Khorshidi G., Douaud G., Beckmann C.F., Glasser M.F., Griffanti L., Smith S.M. (2014). Automatic denoising of functional MRI data: Combining independent component analysis and hierarchical fusion of classifiers. NeuroImage.

[31] Burgess G.C., Kandala S., Nolan D., Laumann T.O., Power J.D., Adeyemo B., Harms M.P., Petersen S.E., Barch D.M. (2016). Evaluation of Denoising Strategies to Address Motion-Correlated Artifacts in Resting-State Functional Magnetic Resonance Imaging Data from the Human Connectome Project. Brain Connect..

[32] Smitha K., Raja K.A., Arun K., Rajesh P., Thomas B., Kapilamoorthy T., Kesavadas C. (2017). Resting state fMRI: A review on methods in resting state connectivity analysis and resting state networks. Neuroradiol. J..

[33] Beckmann C., Smith S. (2004). Probabilistic independent component analysis for functional magnetic resonance imaging. IEEE Trans. Med. Imaging.

[34] Madsen K.H., Churchill N.W., Mørup M. (2017). Quantifying functional connectivity in multi-subject fMRI data using component models. Hum. Brain Mapp..

[35] Leonardi N., Richiardi J., Gschwind M., Simioni S., Annoni J.M., Schluep M., Vuilleumier P., Van De Ville D. (2013). Principal components of functional connectivity: A new approach to study dynamic brain connectivity during rest. NeuroImage.

[36] Caffo B.S., Crainiceanu C.M., Verduzco G., Joel S., Mostofsky S.H., Bassett S.S., Pekar J.J. (2010). Two-stage decompositions for the analysis of functional connectivity for fMRI with application to Alzheimer’s disease risk. NeuroImage.

[37] Calhoun V., Adali T., Pearlson G., Pekar J. (2001). A method for making group inferences from functional MRI data using independent component analysis. Hum. Brain Mapp..

[38] Kolda T.G., Bader B.W. (2009). Tensor decompositions and applications. SIAM Rev..

[39] Beckmann C., Smith S. (2005). Tensorial extensions of independent component analysis for multisubject FMRI analysis. NeuroImage.

[40] Solo V., Poline J.B., Lindquist M.A., Simpson S.L., Bowman F.D., Chung M.K., Cassidy B. (2018). Connectivity in fMRI: Blind Spots and Breakthroughs. IEEE Trans. Med. Imaging.

[41] Simpson S.L., Moussa M.N., Laurienti P.J. (2012). An exponential random graph modeling approach to creating group-based representative whole-brain connectivity networks. NeuroImage.

[42] Simpson S.L., Laurienti P.J. (2015). A two-part mixed-effects modeling framework for analyzing whole-brain network data. NeuroImage.

[43] Bandettini P.A., Jesmanowicz A., Wong E.C., Hyde J.S. (1993). Processing strategies for time-course data sets in functional MRI of the human brain. Magn. Reson. Med..

[44] Marrelec G., Krainik A., Duffau H., Pélégrini-Issac M., Lehéricy S., Doyon J., Benali H. (2006). Partial correlation for functional brain interactivity investigation in functional MRI. NeuroImage.

[45] Podobnik B., Stanley H.E. (2008). Detrended cross-correlation analysis: A new method for analyzing two nonstationary time series. Phys. Rev. Lett..

